# The inflammatory kinase IKKα phosphorylates and stabilizes c-Myc and enhances its activity

**DOI:** 10.1186/s12943-021-01308-8

**Published:** 2021-01-18

**Authors:** Bernhard Moser, Bernhard Hochreiter, José Basílio, Viola Gleitsmann, Anja Panhuber, Alan Pardo-Garcia, Bastian Hoesel, Manuel Salzmann, Ulrike Resch, Mamoona Noreen, Johannes A. Schmid

**Affiliations:** Institute of Vascular Biology and Thrombosis Research, Center for Physiology and Pharmacology, Medical University of Vienna, Schwarzspanierstraße 17, 1090 Vienna, Austria

**Keywords:** c-Myc, IKKα, NF-κB, Cancer, Inflammation

## Abstract

**Background:**

The IκB kinase (IKK) complex, comprising the two enzymes IKKα and IKKβ, is the main activator of the inflammatory transcription factor NF-κB, which is constitutively active in many cancers. While several connections between NF-κB signaling and the oncogene c-Myc have been shown, functional links between the signaling molecules are still poorly studied.

**Methods:**

Molecular interactions were shown by co-immunoprecipitation and FRET microscopy. Phosphorylation of c-Myc was shown by kinases assays and its activity by improved reporter gene systems. CRISPR/Cas9-mediated gene knockout and chemical inhibition were used to block IKK activity. The turnover of c-Myc variants was determined by degradation in presence of cycloheximide and by optical pulse-chase experiments.. Immunofluorescence of mouse prostate tissue and bioinformatics of human datasets were applied to correlate IKKα- and c-Myc levels. Cell proliferation was assessed by EdU incorporation and apoptosis by flow cytometry.

**Results:**

We show that IKKα and IKKβ bind to c-Myc and phosphorylate it at serines 67/71 within a sequence that is highly conserved. Knockout of IKKα decreased c-Myc-activity and increased its T58-phosphorylation, the target site for GSK3β, triggering polyubiquitination and degradation. c-Myc-mutants mimicking IKK-mediated S67/S71-phosphorylation exhibited slower turnover, higher cell proliferation and lower apoptosis, while the opposite was observed for non-phosphorylatable A67/A71-mutants. A significant positive correlation of c-Myc and IKKα levels was noticed in the prostate epithelium of mice and in a variety of human cancers.

**Conclusions:**

Our data imply that IKKα phosphorylates c-Myc on serines-67/71, thereby stabilizing it, leading to increased transcriptional activity, higher proliferation and decreased apoptosis.

**Supplementary Information:**

The online version contains supplementary material available at 10.1186/s12943-021-01308-8.

## Background

Inflammatory processes are mainly mediated via the canonical NF-κB signaling pathway. The nuclear factor κB (NF-κB) family of transcription factors consists of five members: p65 (RelA), RelB, c-Rel, NF-κB1 (p105/p50) and NF-κB2 (p100/p52) building homo- or heterodimers [1], which can bind to specific enhancer elements in the promoters of target genes thereby regulating inflammation and the immune system. Under normal, physiological conditions, NF-κB is kept inactive by binding to inhibitory proteins of the IκB family or by the intrinsic inhibitory domains of the proforms p100 and p105 [2, 3]. For activation of NF-κB, these inhibitory proteins and domains have to be phosphorylated by IκB kinases (IKKs), which mark them for polyubiquitination and proteasomal degradation. IKKs are organized as a protein complex consisting of two kinases, IKKα (IKK1) and IKKβ (IKK2), and the non-catalytic accessory scaffold protein IKKγ (NEMO). Many different stimuli or cellular stress conditions, such as inflammatory cytokines, or components of pathogens, cause an activation of the IKK complex, leading to phosphorylation and degradation of NF-κB inhibiting proteins or domains. This releases the NF-κB dimer and results in a shift of its steady state localization into the nucleus, where it activates a high number of target genes, depending on the cell type and chromatin accessibility [3–6]. Although IKKα and IKKβ are biochemically and structurally related and act in a concerted manner within the IKK-complex, they do not have identical physiological roles and differ in specific molecular functions, which they can exert outside the IKK-complex. This is exemplified by the fact that they can phosphorylate not only IκB molecules, but also other substrates with distinct specificities thereby regulating a multitude of biological functions [7]. In contrast to IKKβ, which is predominantly found in the cytoplasm, IKKα can shuttle to the nucleus, where it is not only implicated to contribute to cell-cycle regulation, cell differentiation or apoptosis, but also to tumor progression and metastasis [8–11]. A truncated, nuclear active form of IKKα (IKKα(p45)), containing the kinase domain and interacting with full-length IKKα was found to phosphorylate histone H3 and nuclear co-repressors independent of canonical NF-κB signaling and to facilitate ATM activation and DNA repair, after being phosphorylated and activated by the MAP kinases BRAF and TAK1 [12, 13]. In keratinocytes, the sub-cellular localization of IKKα determines mechanisms that promote skin tumor development. In the cytoplasm, IKKα increases levels of epidermal growth factor receptor (EGFR), vascular endothelial growth factor A (VEGF-A) and matrix metalloproteinase 9 (MMP-9), while in the nucleus it elevates the level of the proto-oncogene c-Myc, and furthermore delocalizes Integrin-α6 and downregulates Maspin [14]. Recent studies showed that IKKα signaling promotes lung tumor progression, as well as metastasis and increased malignancy of non-small cell lung cancer. It has been shown that nuclear IKKα acts via Smad2/3, Snail and c-Myc activation, while cytoplasmic IKKα activates NF-κB and EGFR to promote lung tumor progression [15]. One report claimed already that IKKα interacts with c-Myc and influences its stability [16]. However, this study used an IKK-inhibitor, which latter turned out to be very unspecific [17] and employed overexpression of IKKα or IKKβ, while we tried to elucidate the functional links between IKKs and c-Myc using CRISPR/Cas9-mediated gene knockout (KO) and further functional assays.

Deregulation of c-Myc contributes to more than 50% of human cancers by increasing cell proliferation and survival, genetic instability, angiogenesis, and metastasis. Hence, MYC is one of the most potent oncogenes [18]. Inflammation and cancer are tightly linked processes, in which NF-κB activation has been identified as a critical link [2, 19]. However, specific interconnections between single components of the NF-κB pathway and tumorigenesis are still not well understood. This is particularly true for IKKα and IKKβ, which have distinct target substrates and exhibit separate and non-redundant biological functions [7].

Here, we show that transcriptional activity of c-Myc is highly dependent on IKKα but not on IKKβ excluding a possible reciprocal upregulation of c-Myc activity by IKKβ through IKKα as it was proposed before [16]. We identified highly conserved IKK target sites at serine-67 and serine-71 of c-Myc (SGLCS) and show that both IKKα and IKKβ can phosphorylate human c-Myc at the N-terminal end. Furthermore, we demonstrate that knockout of IKKα, but not of IKKβ increases threonine-58 phosphorylation, thereby reducing c-Myc stability and activity. We propose a model in which IKKα phosphorylates c-Myc at serine-67 and serine-71 in the nucleus, leading to subsequent inhibition of GSK3β (glycogen synthase kinase 3β) mediated phosphorylation, which is important for degradation of c-Myc, thus stabilizing c-Myc and enhancing its transcriptional activation, thereby increasing proliferation and inhibiting apoptosis.

## Methods

### Cell lines and cell culture

HEK-293, HeLa and DU145 cells were purchased from the American Type Culture Collection (ATCC). All cell lines were routinely maintained in Dulbecco’s modified Eagle’s medium (DMEM) containing 10% fetal bovine serum (FBS), phenol red, 100 U/mL penicillin, 0.1 mg/mL streptomycin and 2 mM glutamine. All cells were cultured at 37 °C with 5% CO_2_. Transient transfections were performed with cells at 70% confluence using TurboFect™ (Thermo Fisher Scientific) according to the manufacturer’s protocol. Cells were treated with TNFα (Bio-Techne) or the IKK-inhibitor BMS-345541 (Sigma-Aldrich), as indicated.

### Generation of CRISPR/Cas9 knockout cell lines

Single guide RNA (sgRNA) sequences for CRISPR/Cas9 were designed using DeskGen (*Desktop Genetics,* http://www.deskgen.com/) using only high-efficiency sgRNAs, avoiding off-target binding in coding regions. The following sgRNA oligonucleotides were used: IKKα: 5′-ACAGACGTTCCCGAAGCCGC-3′ (GeneID: 1147),

IKKβ: 5′-GCTGACCCACCCCAATGTGG-3′ (GeneID: 3551),

c-Myc: 5′-TTTTCGGGTAGTGGAAAACC-3′ (GeneID: 4609).

To generate CRISPR/Cas9 KO plasmids, complementary oligonucleotides for sgRNAs were annealed and ligated into the BbsI-digested (New England Biolabs) pSpCas9(BB)-2A-Puro (PX459) V2.0 (Addgene plasmid #62988, Addgene) [20] plasmid. All sequences were verified by sequencing prior to experimental use. Cells were transfected with pSpCas9(BB)-2A-Puro (PX459) V2.0 [20] bearing respective sgRNA inserts, followed by treatment with 2–4 μg/ml of puromycin 1 day after transfection for further 48 h. After ~ 2 weeks, cell colonies were isolated using glass cloning cylinders (Sigma-Aldrich) and successful genome edits were analyzed by DNA sequencing, Western blot and quantitative PCR (qPCR).

### Expression constructs

c-Myc in pcDNA3 was a kind gift of Wafik El-Deiry [21]. It was then cloned via BamHI and XbaI (both New England Biolabs) into pEGFP-C1, p-EYFP-C1 and pmDsRed-C1 from Clontech (now Takara Bio). Dendra2-tagged c-Myc was created by replacing pmDsRed for Dendra2 (obtained from pDendra2-B from S. Jakobs) with NheI and BamHI (both New England Biolabs). Mutants of c-Myc were generated with the QuikChange mutagenesis kit (Stratagene) to replace serines 67 and 71 with alanines (c-MycAA, non-phosphorylatable at these sites) or with glutamate (c-MycEE, mimicking permanent phosphorylation). IKKα tagged with mCherry was cloned from EYFP-IKKα [11], by exchanging EYFP for mCherry. All plasmids were checked by sequencing before use. To generate stable transfectants, c-Myc knockout DU145 cells were transfected with c-Myc, c-MycAA or c-MycEE expression plasmids and treated with 800 ng/ml of geneticin G418 (Carl Roth, Karlsruhe, Germany) to select positive clones.

### Co*-*immunoprecipitation

10^7^ HeLa cells were washed once with Dulbecco’s PBS, scraped of the tissue culture dish and lysed in lysis buffer (20 mM Tris HCL pH 8.0; 137 mM NaCl; 10% glycerol; 1% NP-40; 2 mM EDTA; Protease Inhibitor Cocktail, Roche). Then, the cell lysate was treated with 10 U/μL Benzonase (Sigma-Aldrich) and incubated for 1 h at 4 °C. Afterwards, anti-IKKα (#61294, Cell Signaling Technology) or anti-IKKβ (#8943, Cell Signaling Technology) antibodies were incubated with the cell lysates for 16 h at 4 °C under rotation. To pull down antibody-protein-protein complexes, magnetic Dynabeads (Thermo Fisher Scientific) were added and incubated for further 4 h at 4 °C under rotation. Subsequently, the magnetic beads were collected, washed three times with lysis buffer and subjected to Western blot analysis with the antibodies indicated in the figures.

### Flow cytometry

To characterize cells stably expressing c-Myc, c-MycAA and c-MycEE, cells were trypsinized, washed and labeled with Annexin V-APC (Biolegend) and 7-Aminoactinomycin D (7-AAD, Thermo Fisher Scientific) in Annexin V binding buffer (Biolegend) for 15 min at room temperature. All samples were analyzed without washing using a CytoflexS flow cytometer (Beckman-Coulter) with CytExpert 2.4 software.

### Quantification of DNA-synthesis by EdU incorporation

DU145 cells stably transfected with the c-Myc mutants c-MycAA or c-MycEE, or HEK-293 cells transiently transfected with c-Myc, c-MycAA or c-MycEE were seeded on 96-well plates and incubated at 37 °C with 6 μM EdU (5-ethynyl-2′-deoxyuridine), a nucleoside analog similar to BrdU for 4 h. EdU incorporated into the DNA of proliferating cells was labeled by click-chemistry with 5-TAMRA-PEG3-Azide (base-click kit, BCK-HTS555, Sigma-Aldrich) and quantified using a microplate fluorescence reader (Synergy H4, BioTek, excitation at 546 nm and emission at 580 nm).

### Gene expression analysis

The Cancer Genome Atlas (TCGA) datasets (https://www.cancer.gov/) were extracted using the commercial tool Genevestigator (v.7.6.2) [22]. The R package ggpubr (v.0.4.0, https://CRAN.R-project.org/package=ggpubr) was used to calculate the CHUK (IKKα)/MYC (c-Myc) Pearson’s correlation coefficient and respective *p*-value, and to add the regression line equation. The plot was done with ggplot2 (v.3.3.2) [23].

### Immunofluorescence staining of prostate sections from transgenic mice

Mice expressing elevated levels of c-Myc, specifically in the prostate epithelium (Hi-MYC, [24]), which develop prostate cancer, were compared with wild-type controls of the same genetic background (C57BL/6 J). Prostate tissue was processed with standard histology routines to generate paraffin sections. These were deparaffinized and hydrated using a standard xylene-ethanol series. Antigen retrieval was done in 10 mM citrate buffer. After washing with Tris-buffer salt solution, 0.1% Triton X-100 (TBST), sections were blocked with 1% FBS in TBST and incubated with antibodies overnight at 4 °C. The following antibodies were used: rabbit anti-c-Myc/N-Myc (#13987, Cell Signaling Technology), mouse anti-IKKα (sc-7606, Santa Cruz) and as secondary antibodies: donkey anti-rabbit IgG H&L, DyLight® 650 (ab96922, Abcam) and donkey anti-mouse IgG H&L, Dylight 550 (ab98795, Abcam), as specified in the key resources table. Nuclei were counterstained with DAPI. Stained sections were imaged with a Nikon A1 R+ confocal microscope and analyzed as described below.

### Kinase assay

HEK-293 cells transfected with flag-tagged IKKα, IKKβ or constitutive active variants thereof (comprising glutamates at position S177/S181) were lysed in kinase lysis buffer containing 20 mM Tris/HCl pH 7.5, 150 mM NaCl, 25 mM β-glycerophosphate, 2 mM EDTA, 2 mM pyrophosphate, 1 mM orthovanadate, 1% Triton X-100, 1 mM DTT, 1 mM NaF and cleared by centrifugation. Flag-tagged kinases were immunoprecipitated with anti-flag affinity matrix beads (Thermo Fisher Scientific) and washed three times with PBS and once with kinase buffer (20 mM Tris/HCl pH 7.5, 20 mM β-glycerophosphate, 100 μM orthovanadate, 10 mM MgCl_2_, 50 mM NaCl, 1 mM DTT, 1 mM NaF). Then, 5 nmol of biotinylated peptide substrate (obtained from GenScript, sequences as indicated in Fig. 2), were added to the beads containing kinases in 10 μl kinase buffer supplemented with ^32^P-γ-ATP (5 μCi per sample) and 10 mM MnCl_2_ and incubated at 30 °C for 2 h. The reaction mixtures were transferred to NeutrAvidin-coated strips (Pierce, Thermo Scientific) to bind the biotinylated peptides (2 h at room temperature), followed by 10 times washing with TBST and release of ^32^P by incubation with 1 M NaOH at 55 °C for 1 h. The released ^32^P was quantified by liquid scintillation counting employing a ^32^P- specific protocol. For detection of phosphorylation of full-length c-Myc, 1 μg of recombinant protein (RayBiotech) was added to constitutively active flag-tagged IKKα bound to flag-affinity matrix under conditions as above, followed by SDS-PAGE on a 20% homogenous PhastGel (Thermo Fisher Scientific), drying of the gel and exposure on x-ray film for 24 h. The gel-detection method as described above was also applied to non-biotinylated peptides (as shown in Fig. 2c).

### Microscopic analysis

FRET microscopy and imaging of tissue was done on a Nikon A1 R+ laser scanning confocal system using a 60x plan apochromatic oil immersion objective (NA1.4). FRET-microscopy was done as previously described [25, 26] (for the Figure S1 as described in [27]). Spectral scan images of mouse prostate sections were acquired with four lasers: 405 nm, 488 nm, 561 nm and 650 nm in a range between the laser wavelength and an upper limit of 760 nm with a spectral bin size of 10 nm. Image analysis was done in the free *ImageJ* software package *Fiji* (https://fiji.sc/). Specific fluorescence signals and background were separated by linear spectral unmixing. Regions of the prostate epithelium were determined by thresholding and separated into smaller regions by watershedding. Intensities were measured and for further analysis normalized to the nuclear stain (DAPI) signal to account for variations in slice thickness and cell density. Used code will be freely shared upon reasonable request.

### Optical pulse-chase experiments with Dendra2

c-Myc, as well as the phosphorylation mutants c-MycAA (non-phosphorylatable) and c-MycEE (mimicking phosphorylated c-Myc) were cloned into an expression construct as fusion proteins with the photoconvertible fluorescent protein Dendra2 [28, 29]. HEK-293 cells on glass coverslips were transfected with these constructs and imaged at 37 °C on a life-cell microscope (Olympus IX71) equipped with a temperature- and 5% CO_2_ control chamber and a monochromator light source (Polychromator IV, TILL Photonics). Photoconversion of Dendra2 from green to red fluorescence was achieved by 50 cycles of 1-s illumination at 405 nm (which had been optimized before). Thereafter, images of the red fluorescence were taken every 15 min for 4 h. Decrease of the red fluorescence as quantified with ImageJ indicated degradation of the photoconverted protein, as newly synthesized protein is always green fluorescent.

### Determination of protein turnover using cycloheximide

Stable transfectants of DU145 cells expressing c-MycAA or c-MycEE or HEK-293 cells transiently transfected with c-Myc mutants were incubated for different time periods with the protein synthesis inhibitor cycloheximide at a concentration of 60 μg/ml. Thereafter, cells were lysed in presence of protease inhibitors (Complete™, Roche), followed by SDS-PAGE and Western Blotting for c-Myc using CCD-camera based detection of luminescence and quantification of c-Myc bands by *ImageJ*.

### Reporter gene assay

The c-Myc dependent CDK4 (Gene ID: 1019) promoter was derived by PCR using a Q5 High-Fidelity DNA Polymerase (New England Biolabs) and the following primer pairs:

5′-ATATTAGCTAGCGGGTTGTGGCAGCCAGTCA-3′; and 5′-TATTAAAGCTTCGAACGC CGGACGTTCTG-3′ (Annealing temperature: 68 °C). The CDK4 promoter region was released by the use of NheI and HindIII (both New England Biolabs) and ligated in the pNL1.1 Nanoluc Reporter vector (Promega). DU145 cells were transiently transfected with this reporter construct in combination with a constitutively expressing β-galactosidase plasmid, driven by a ubiquitin-promoter: PUB6/V5-His/LacZ (Thermo Fisher Scientific), which was used as an internal standard to normalize luciferase activity. Cells were lysed in a passive lysis buffer (0.1 MKH_2_PO_4_, and 0.1% Triton X-100) and Nanoluc luminescence was subsequently measured according to product specifications of Nano-Glo® Luciferase Assay System (Promega) and normalized to β-galactosidase activity. The latter was determined by incubation with chlorophenol red-β-D-galactopyranoside as substrate (CPRG: 1 mg/ml in PBS, 10 mM KCl, 1 mM MgCl_2_) and detection at 570 nm with a standard ELISA reader.

### Western blotting

Cells were lysed in Laemmli Buffer (50 mM Tris-HCl, 10% Glycerol, 10% SDS, 0.2% Bromophenol blue, 5% β-Mercaptoethanol) and proteins were denatured for 10 min at 95 °C. Equal protein amounts were separated by SDS-PAGE using 10% polyacrylamide. Proteins were analyzed after blotting onto PVDF membranes (Carl Roth, Germany), which were blocked with 5% Skim Milk Powder (Sigma-Aldrich) in TBS containing 0.1% Tween20. All primary antibodies were incubated overnight at 4 °C with the PVDF membranes; followed by incubation with secondary antibodies for 1 h at room temperature. Proteins were detected using Western Bright Chemiluminescence Substrate Sirius (Biozym), visualized by FluorChem HD2 Chemiluminescence Imager and quantified with *ImageJ*. The antibodies used were: anti-GAPDH (#5174), anti-IKKα (#11930), anti-IKKβ (#8943), anti-c-MYC (#13987), anti-GSK-3β (#12456), anti-rabbit IgG, HRP-linked antibody (#7074), anti-mouse IgG, HRP-linked antibody (#7076) (all Cell Signaling Technology).

### Statistical analysis

Data were statistically evaluated by using GraphPad Prism 8.01 software and depicted as bar graphs indicating mean ± standard deviation (SD). Statistical significances are depicted as: * *P* ≤ 0.05, ** *P* ≤ 0.01, *** *P* ≤ 0.001, **** *P* ≤ 0.0001.

### Key resources table

**Table:** 

CHEMICALS AND REAGENTS		
BMS-34A3:C22	Sigma-Aldrich, Vienna, Austria	Cat#: B9935
^32^P-γ-ATP	Hartmann Analytic, Braunschweig, Germany	Cat#: SRP-301H
ANTI-FLAG M2 AFFINITY GEL	Thermo Scientific, Vienna, Austria	Cat#: A2220
Benzonase, Nuclease	Sigma-Aldrich, Vienna, Austria	Cat#: E1014-5KU
CFSE Cell Division Tracker Kit	BioLegend, CA, USA	Cat#: 423801
cOmplete,Protease Inhibitor Cocktail	Roche, Vienna, Austria	Cat#: 11836170001
Dynabeads	Thermo Scientific, Vienna, Austria	Cat#: 1002D
EdU HTS Kit 555	Sigma-Aldrich, Vienna, Austria	Cat#: BCK-HTS555
FxCycle Violet Stain	Thermo Scientific, Vienna, Austria	Cat#: F10347
Nano-Glo Luciferase Assay System	Promega, Mannheim, Germany	Cat#: N1110
NeutrAvidin-coated strips	Pierce, Thermo Scientific, Vienna, Austria	Cat#: 15127
PhastGel	Thermo Scientific, Vienna, Austria	Cat#: 10734927
PVDF-Membran	Roth, Karlsruhe, Germany	Cat#: T830.1
Pyrex(R) cloning cylinder	Sigma-Aldrich, Vienna, Austria	Cat#: CLS31666-125EA
QuikChange mutagenesis kit	Stratagene, Santa Clara, CA	Cat#: 200518
Recombinant Human Proto-oncogene c-Myc	RayBiotech, Georgia, USA	Cat#: RB-230-00580-50
Skim Milk Powder	Sigma-Aldrich, Vienna, Austria	Cat#: 70166
SYTOX Green Nucleic Acid Stain	Thermo Scientific, Vienna, Austria	Cat#: S7020
TNFα	Bio-Techne Ltd., Minnesota, USA	Cat#: 210-TA-020
TurboFect	Thermo Scientific, Vienna, Austria	Cat#: R0531
WesternBright Chemilumineszenz Substrat	Biozym, Vienna, Austria	Cat#: 541021
ANTIBODIES		
Anti-mouse IgG, HRP-linked Antibody (1:1000)	Cell Signaling Technology, Frankfurt, Germany	Cat#: 7076; RRID: AB_330924
Anti-rabbit IgG, HRP-linked Antibody (1:1000)	Cell Signaling Technology, Frankfurt, Germany	Cat#: 7074; RRID: AB_2099233
APC Annexin V (1:50)	BioLegend, CA, USA	Cat#: 640920
c-Myc/N-Myc (D3N8F) Rabbit mAb (1:1000)	Cell Signaling Technology, Frankfurt, Germany	Cat#: 13987; RRID: AB_2631168
Donkey Anti-Mouse IgG H&L (Dylight 550) (1:200)	Abcam, Cambridge, UK	Cat#: ab98795; RRID: AB_10675196
Donkey Anti-Rabbit IgG H&L (DyLight 650) (1:200)	Abcam, Cambridge, UK	Cat#: ab96922; RRID: AB_10680408
GAPDH (D16H11) XP® Rabbit mAb (1:1000)	Cell Signaling Technology, Frankfurt, Germany	Cat#: 5174; RRID: AB_10622025
GSK-3β (D5C5Z) XP® Rabbit mAb (1:1000)	Cell Signaling Technology, Frankfurt, Germany	Cat#: 12456; RRID: AB_2636978
IKKα (3G12) Mouse mAb (1:1000)	Cell Signaling Technology, Frankfurt, Germany	Cat#: 11930; RRID: AB_2687618
IKKα (B-8) mouse (1:50)	Santa Cruz, CA, USA	Cat#: sc-7606; RRID: AB_627784
IKKα (D3W6N) Rabbit mAb (1:200)	Cell Signaling Technology, Frankfurt, Germany	Cat#: 61294; RRID: AB_2799606
IKKβ (D30C6) Rabbit mAb (IF 1:100, WB 1:1000)	Cell Signaling Technology, Frankfurt, Germany	Cat#: RRID: AB_11024092
Phospho-c-Myc (Ser62) Polyclonal, IgG, Rabbit (1:200)	Amsbio, Abington, UK	AMS.E-AB-21265
Phospho-c-Myc (Thr58) Polyclonal, IgG, Rabbit (1:200)	Amsbio, Abington, UK	AMS.E-AB-20845
ENZYMES		
BamHI	New England Biolabs, Frankfurt, Germany	Cat#: R3136
BbsI	New England Biolabs, Frankfurt, Germany	Cat#: R3539
HindIII	New England Biolabs, Frankfurt, Germany	Cat#: R3104
NheI	New England Biolabs, Frankfurt, Germany	Cat#: R3131
XbaI	New England Biolabs, Frankfurt, Germany	Cat#: R0145
Q5 High-Fidelity DNA Polymerase	New England Biolabs, Frankfurt, Germany	Cat#: M0491S
PLASMIDS		
pNL1.1 [Nluc] Vector	Promega, Mannheim, Germany	Cat#: N1001
pcDNA3	Invitrogen, A, USA	Cat#: discontinued
pEGFP-C1	Clonetech (now Takara Bio), CA, USA	Cat#: discontinued
pEYFP-C1	Clonetech (now Takara Bio), CA, USA	Cat#: discontinued
pmDsRed-C1	Clonetech (now Takara Bio), CA, USA	Cat#: discontinued
pSpCas9(BB)-2A-Puro (PX459) V2.0	Addgene, Massachusetts, USA	Cat#: 62988; RRID: Addgene_62,988
PUB6/V5-His/LacZ	Thermo Scientific, Vienna, Austria	Cat#: V25020
SOFTWARE		
CytExpert	Beckman Coulter, Vienna, Austria	RRID:SCR_017217
Genevestigator	Nebion AG, Zurich, Switzerland	RRID:SCR_002358
Graphpad Prism	GraphPad Software, CA, USA	RRID:SCR_002798
ImageJ/Fiji	Open source	RRID: SCR_003070

## Results

### IKKα directly interacts with c-Myc in the nucleus

**Fig. 1:**
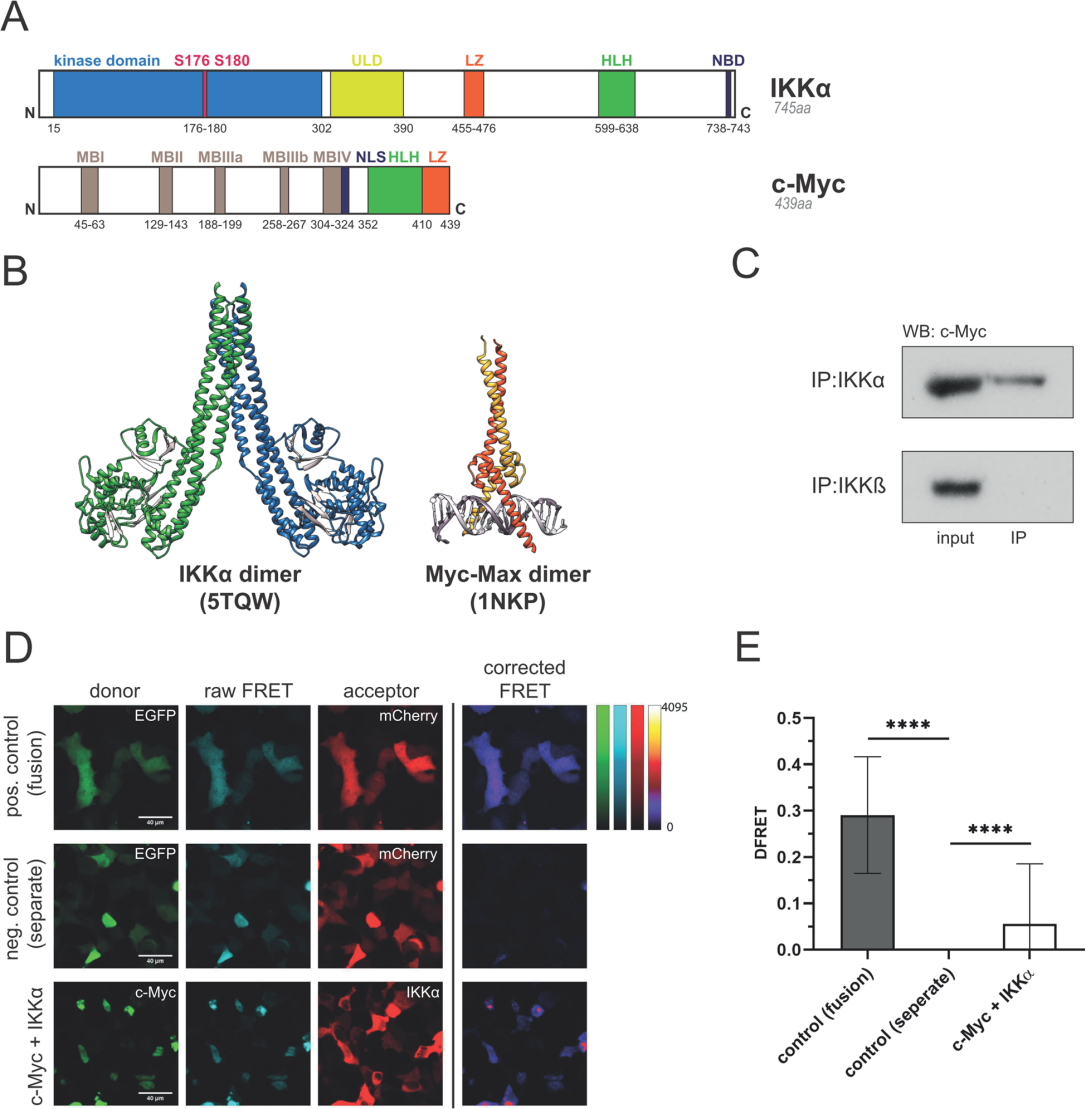
IκB kinase α (IKKα) directly interacts with c-Myc. **a** Schematic illustration of IKKα and c-Myc domains: NBD: NEMO binding domain; ULD: ubiquitin-like domain; MBI – MBIV: Myc boxes; NLS: nuclear localization signal (dark blue); HLH: helix-loop-helix domain; LZ: leucine zipper. Positions of amino acids are indicated by numbers. **b** 3D- models of an IKKα homodimer (PDB-code: 5TQW) and a c-Myc-Max heterodimer bound to DNA (PDB-code: 1NKP), depicting comparable protein-interaction domains at the coiled HLH/LZ regions. **c** Interaction between IKKα and c-Myc was shown by co-immunoprecipitation. Either IKKα or IKKβ were immunoprecipitated (IP) from DU145 cell extracts (input), followed by SDS-PAGE and Western blot (WB) to detect c-Myc. **d** Verification of the IKKα/c-Myc interaction by FRET microscopy: Expression constructs of an EGFP-mCherry fusion protein (positive control, fusion), unbound EGFP and mCherry (negative control, separate proteins), EGFP-tagged c-Myc and mCherry-tagged IKKα fusion proteins, were transfected into HEK-293 cells as indicated. 1d after transfection FRET microscopy was performed as described in detail in the Methods section. Donor channel: EGFP excitation and emission); acceptor channel: mCherry excitation and emission; raw FRET channel: EGFP excitation and mCherry emission. The corrected FRET images show the FRET signal after subtraction of the spectral bleed-through (according to Youvan et al. [30]) and is a qualitative assessment of the interaction without normalization to expression levels). **e** Quantitative assessment of normalized FRET values based on corrected donor fluorescence (DFRET) as described in [26]. Statistical analysis was performed using two-way ANOVA and Dunnett’s multiple comparison, *p*-values: ‘****’ for *p* < 0.0001. Error bars represent mean ± standard deviation (*n* = 508 for the fusion control; *n* = 236 for the separate control and *n* = 394 for c-Myc + IKKα)

IKKα and IKKβ exhibit high similarities in their structure both containing a NH_2_-terminal protein kinase domain and leucine zipper (LZ) and helix-loop-helix (HLH) c*arboxy*-*terminal* motifs, while showing several differences in their molecular functions and kinase activities [7]. In contrast to IKKβ, IKKα constantly shuttles between the nucleus and the cytoplasm [11] and is recruited to specific promoters of NF-κB target genes, where it phosphorylates distinct substrates to regulate gene expression [8, 9]. The predominantly nuclear oncoprotein c-Myc comprises a basic HLH motif (Fig. 1a) and interacts with other HLH-LZ containing proteins, like the Myc-associated factor (Max). This dimerization, the subsequent DNA-binding and the oncogenic activity of c-Myc and Max proteins require the basic HLH-LZ motif [31]. Since HLH-LZ domains often serve as interaction motif and based on structural similarities between IKK- and c-Myc/Max dimers (Fig. 1b), we hypothesized that IKKs might interact with c-Myc via this domain, providing a molecular link between inflammatory signaling molecules and the central oncogene c-Myc, which is upregulated in a high percentage of cancers. The structural similarities prompted us to test for a direct interaction between c-Myc and IKKα. To that end, we applied two independent methods: i) co-immunoprecipitation followed by Western blot analysis and ii) fluorescence resonance energy transfer (FRET) microscopy. Co-immunoprecipitation clearly revealed an interaction between c-Myc and IKKα but no detectable association with IKKβ (Fig. 1c). Next, we wanted to determine the subcellular localization of the interaction. To that end, we applied FRET microscopy, which is ideally suited to monitor close proximities and thus macromolecular binding in living cells. It has the advantage over co-immunoprecipitation that it can reveal not only whether two proteins bind to each other, but furthermore the subcellular site of the interaction. This experimental approach demonstrated that IKKα and c-Myc interact with each other within the nucleus (Fig. 1d) and also showed a weak signal between c-Myc and IKKβ (Suppl. Figure S1), which can be explained by a lower degree of colocalization of these two molecules. In order to obtain a robust quantitative assessment of the interaction between IKKα and c-Myc, we used a more sophisticated method with titration of different acceptor to donor ratios [26]. Calculation of a normalized and corrected FRET efficiency value (DFRET as it is based on normalization to the donor), verified the statistical significance of this interaction (Fig. 1e).

### c-Myc is phosphorylated by IKKs on serine-67 and -71

**Fig. 2:**
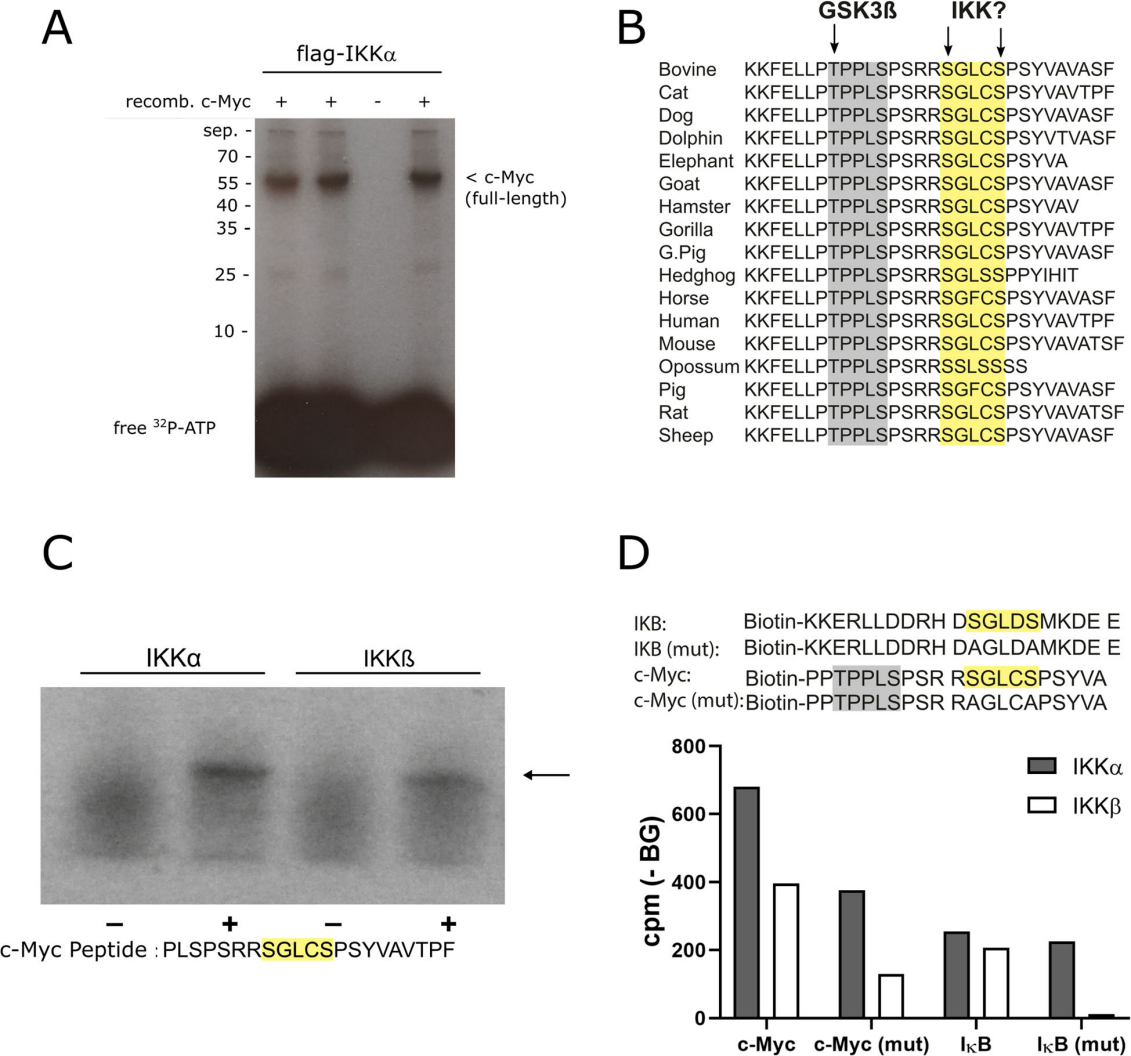
IκB kinases phosphorylate c-Myc at the N-terminal end. **a** Phosphorylation of full-length c-Myc by IKKα: HEK-293 cells were transfected with a flag-tagged constitutive active IKKα expression construct, followed by purification of the kinase with anti-flag beads and an in vitro kinase reaction with recombinant c-Myc. After the kinase reaction, samples were applied to SDS-PAGE and the dried gel was exposed to an x-ray film. Three independent samples with the substrate (+) are shown in comparison to a negative control (−). Unconjugated, free ^32^P-ATP is seen at the front of the gel. **b** Protein alignment showing conservation of a putative IKK-target site (SGLCS motif) next to the known GSK3β phosphorylation site in diverse species. **c** Kinase assay showing IKKα and IKKβ-mediated phosphorylation of a c-Myc peptide (as specified below the gel) containing the putative IKK target site. **d** Peptide kinase assay with detection of the incorporated ^32^P using a liquid scintillation counter. Flag-tagged IKKα or IKKβ was transfected into HEK-293 cells, followed by purification with flag-affinity matrix. Biotinylated peptides of IκB containing a known IKK phosphorylation site or a mutated, negative control (IκB-mut) and peptides containing the putative phosphorylation site of c-Myc or a mutated variant thereof (c-Myc-mut, sequences as indicated) were incubated with the kinases, followed by purification of biotinylated peptides on neutravidin-coated plates and measurement by scintillation counting

The substrate spectrum of IKKα and IKKβ is not restricted to IκB molecules and the homologous NF-κB precursor domains, which are phosphorylated at two distinct serine residues in the SGXXS motif but includes a variety of other proteins [31]. Both kinases have been shown to phosphorylate several tumor suppressor proteins, cell cycle regulators or proteins, which are significantly implicated in signaling pathways such as the Wnt- or the MAPK pathway. To explore whether c-Myc might be a substrate of IKKs, we performed a radioactive kinase assay using immune-purified IKKα and recombinant full-length c-Myc. This revealed prominent phosphorylation of c-Myc with a single band at the expected molecular weight (Fig. 2a). Next, we screened the amino acid sequence of human c-Myc for sites with homology to the SGXXS motif and found a hypothetical IKK phosphorylation site, close to the GSK3β target site, at serine-67 and serine-71 (SGLCS), which is very similar to the IKK target site in IκBα (32-SGLDS-36) [32], and which is conserved in many species (Fig. 2b). To evaluate this putative phosphorylation site, we performed a kinase assay with a peptide substrate comprising that region. Both, IKKα and IKKβ, were able to phosphorylate this peptide as revealed by SDS-PAGE and autoradiography (Fig. 2c). Furthermore, we established a kinase assay using biotinylated peptides and tested variants of the putative substrate site in which the target serines were replaced by alanines. For quantification of the enzyme reaction, we purified the phosphorylated peptides on avidin-coated plates, followed by alkaline release of the phosphate and scintillation counting. This experimental approach revealed slightly higher ^32^P-incorporation with IKKα as compared to IKKβ and a radioactivity signal, which was even higher than that of a canonical IκBα peptide of the same length and with the serine residues at the homologous positions. Interestingly, the mutant c-Myc peptide with alanines replacing the putative serine-phosphorylation sites still incorporated some radioactivity upon incubation with IKKα or IKKβ. This mutant peptide contained one threonine and three serine residues in addition to the mutated residues, which might serve as further target sites for IKKs (PPTPPLSPSRRAGLCAPSYVA) (Fig. 2d).

### IKKα- but not IKKβ knockout affects phosphorylation of c-Myc at threonine-58

The stability of c-Myc is regulated by phosphorylation at serine-62 and threonine-58. Serine-62 phosphorylation leads to an intermediate stabilization of c-Myc, while the subsequent phosphorylation of threonine-58 by GSK3β and dephosphorylation of serine-62 leads to its proteasomal degradation [33, 34].

**Fig. 3:**
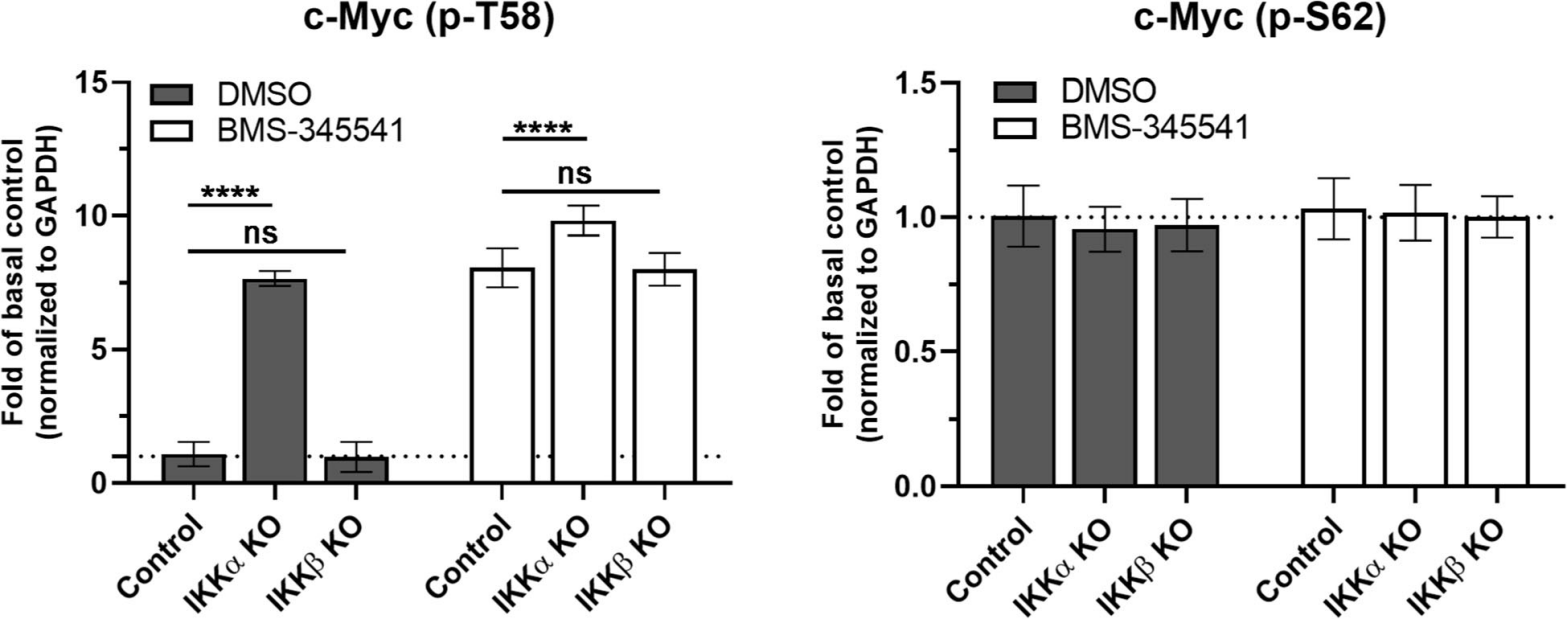
CRISPR/Cas9-mediated knockout of IKKα impairs phosphorylation of c-Myc at threonine-58. Quantification of Western blots (Figure S1) showing extracts of control, IKKα- or IKKβ knockout (KO) cells in absence or presence of the IKK-inhibitor BMS-345541. Deletion of IKKα or treatment with BMS-345541, but not IKKβ knockout significantly increased threonine-58 phosphorylated c-Myc levels while threonine-62 c-Myc phosphorylation was unaffected; *n* = 4. Statistical analysis was performed using two-way ANOVA and Tukey’s multiple comparison. *p*-values: ‘****’ for *p* < 0.0001. Bar graphs represent mean ± standard deviation

To investigate a possible effect of IKKα- or IKKβ-mediated phosphorylation on c-Myc, we analyzed threonine-58 and serine-62 phosphorylation of c-Myc after CRISPR/Cas9 mediated knockout of IKKα or IKKβ. In addition, we tested a potential effect of IKKs by treating prostate cancer cells with the specific IKK inhibitor BMS-345541 at a concentration, where it blocks both IKKα and IKKβ activity. While phosphorylation of serine-62 was unaffected, threonine-58 phosphorylation of c-Myc was significantly increased in IKKα knockout cells and upon inhibition of both IKKs with BMS-345541 (Fig. 3 and suppl. Figure S2). CRISPR/Cas9-mediated knockout showed that only deletion of IKKα, but not IKKβ increased threonine-58 phosphorylation – similar to the effect of an inhibitor targeting both kinases. In summary, these findings indicate that IKKα phosphorylates c-Myc at least on serines 67 and 71, which affects threonine-58 phosphorylation and subsequent degradation, thereby increasing c-Myc protein stability.

### IKKα increases the transcriptional activity of c-Myc and the IKK-target site influences the turnover of c-Myc

Besides its ability to regulate gene expression by remodeling chromatin via histone H3 phosphorylation [8, 9], IKKα can also directly phosphorylate and thus regulate the activity of various transcription factors and co-activators [35–38].

**Fig. 4:**
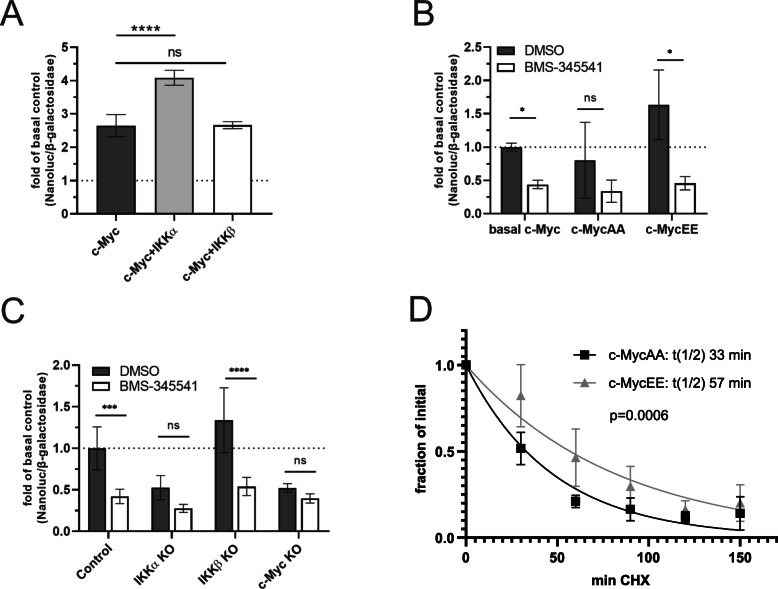
IKKα, but not IKKβ increases the transcriptional activity of c-Myc and dependence of c-Myc stability on the IKK-target site. Transcriptional activity of c-Myc measured with a Nano-luciferase (Nanoluc) reporter gene construct under the control of the c-Myc dependent CDK4 promoter in DU145 cells. **a** Co-transfection of cells with expression constructs of IKKα or IKKβ as indicated; *n* = 5. **b** Transcriptional activity of a phosphorylation-deficient c-Myc mutant (c-MycAA) and a phosphomimicking c-Myc mutant (c-MycEE) in presence or absence of the IKK-inhibitor BMS-345541; *n* = 4. **c** c-Myc activity in control-, IKKα-, IKKβ- and c-Myc knockout cells upon treatment with DMSO (control) or BMS-345541; *n* = 4. **d** Stable DU145 transfectants expressing c-MycAA or c-MycEE were cultured for different time periods in presence of 60 μg/ml cycloheximide to block protein synthesis, followed by cell lysis, SDS-PAGE and Western blotting for c-Myc. The c-Myc-specific bands were quantified by *ImageJ* and subject to single exponential decay curve fitting. Statistical analysis was performed with GraphPad Prism using **a**: one-way ANOVA and Tukey’s post hoc test, (**b**, **c**): two-way ANOVA and Tukey’s multiple comparison and (**d**): curve fitting with a one-phase exponential decay equation using a constraint of 100% as initial value and a constraint of 0% for the plateau. *p*-values: ‘*’ for *p* < 0.05; ‘***’ for *p* < 0.001; ‘****’ for *p* < 0.0001. Mean values are plotted with error bars indicating ± standard deviation

To determine how IKKα influences the transcriptional activity of c-Myc, we used a highly sensitive *CDK4* gene promoter NanoLuc reporter system. Co-transfection of this reporter with a plasmid expressing IKKα significantly increased the transcriptional activity of c-Myc, compared to cells transfected with an IKKβ-expression construct or the basal c-Myc level (Fig. 4a). To investigate the role of the serine-67/71 phosphorylation site in this context, we generated a phosphorylation-deficient c-Myc mutant by changing serine-67 and serine-71 into alanines (c-MycAA) and furthermore we created a mutant mimicking permanent serine-phosphorylation by replacing the serines with negatively charged glutamate residues (c-MycEE). The c-MycAA mutant showed only basal c-Myc activity, while the phosphomimicking c-MycEE mutant exhibited significantly increased transcriptional activity, supporting the significance of this IKK target site for the activity of c-Myc (Fig. 4b). To investigate, whether this effect is specific for IKKα, we performed the c-Myc reporter gene assay using CRISPR/Cas9 mediated knockout cells. Deletion of IKKα but not of IKKβ significantly decreased the transcriptional activity of c-Myc (Fig. 4c).

Furthermore, we tested the hypothesis that phosphorylation of the IKK target site affects stability and turnover of c-Myc. To that end, we generated stable transfectant cells expressing non-phosphorylatable c-MycAA or the phosphomimetic variant c-MycEE and blocked protein synthesis in these cells for different times with cycloheximide, followed by SDS-PAGE and Western Blot detection. Previous studies have shown that this approach is comparable to radioactive pulse/chase experiments using ^35^S-methionine incorporation into newly synthesized proteins [39]. Quantification of the c-Myc bands revealed that the non-phosphorylatable variant c-MycAA was degraded significantly faster than the phosphomimetic variant c-MycEE (Fig. 4d and suppl. Figure S3A). Furthermore, we used an additional approach, where wild-type c-Myc, as well as the phosphorylation-deficient c-MycAA and the phosphomimetic c-MycEE were linked to the fluorescent protein Dendra2, which can be converted from a green to a red fluorescent state upon intense illumination with blue light. This allows optical pulse-chase experiments, as the fusion protein can be switched at a given time point from green to red, while all subsequently synthesized proteins again will be green. Following the decay of the red fluorescence enables an assessment of the degradation kinetics and thus an estimate of the half-life of the protein. Using this technique with transiently transfected HEK-293 cells, we found that c-MycEE had the longest half-life, while c-MycAA showed the fastest degradation kinetics with wild-type c-Myc being in between (Suppl. Figure S3B). However, we noticed that the half-lives of the fusion proteins of c-Myc variants with Dendra2 were longer than those seen in the cycloheximide experiment. This might be due to the transient transfection generating higher levels of expression as compared to the stable transfectants; or alternatively it might be the result of the N-terminal fluorescent protein tag. Nevertheless, within a given system, c-MycEE always exhibited a longer half-life than the mutant c-MycAA.

### Correlation of IKKα and c-Myc expression in mouse prostates and human samples

**Fig. 5:**
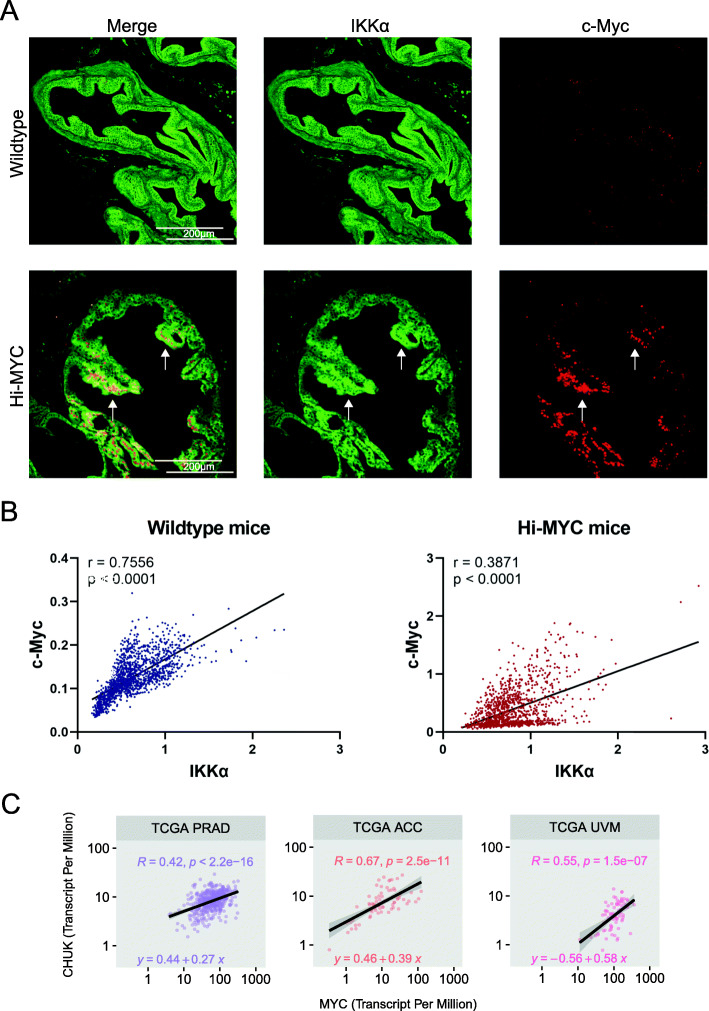
Correlation of c-Myc and IKKα expression in mouse prostates and human cancers. **a** Representative immunofluorescence staining of IKKα and c-Myc in mouse prostate tissues of wild-type and Hi-MYC mice. White arrows indicate regions of coincident high c-Myc/high IKKα levels. **b** c-Myc levels plotted against IKKα expression for wild-type and Hi-MYC prostate sections. Each dot represents a small region of the prostate epithelium normalized to the nuclear stain (DAPI). Note the different y-axis scale. **c** Pearson’s correlation between the expression values in Transcript Per Million of IKKα (CHUK) and c-Myc (MYC) in several types of human cancers from the TCGA dataset. The plot scale was transformed to a log10 scale. PRAD: Prostate adenocarcinoma; ACC: Adrenocortical carcinoma; UVM: Uveal Melanoma; Lines represent the linear regression analysis; r is the Spearman correlation coefficient, p-values indicate statistical significance of positive correlation according to Spearman

c-Myc significantly drives tumorigenesis and increases disease severity in prostate cancer, which correlates with overexpression of c-Myc mRNA and protein [40, 41]. To clarify, whether the expression of c-Myc is associated with IKKα expression in vivo, we investigated a well-established transgene mouse model of prostate cancer, where c-Myc is overexpressed specifically in prostate epithelium (Hi-MYC mouse [24]). Prostate sections of these transgene mice were double-stained by immune-fluorescence for IKKα and c-Myc and compared to wild type controls (Fig. 5a). Intensities of the IKKα- and c-Myc signals of the prostate epithelium regions were quantified and normalized to the nuclear stain (DAPI) to account for variations in slice thickness and cell density. Statistical analysis revealed that c-Myc expression correlated significantly with IKKα levels in wild-type mice (r = 0.7556, *p* < 0.0001) as well as in Hi-MYC mice (r = 0.3871, *p* < 0.0001) (Fig. 5b). Next, we investigated whether a similar correlation can also be observed in human cancer. To that end, we analyzed publicly accessible data from *The Cancer Genome Atlas*, TCGA (https://portal.gdc.cancer.gov/) using the R-package ggpubr. This revealed a significant positive correlation between IKKα (gene symbol: CHUK) and MYC expression in prostate-, as well as other cancer types (Fig. 5c) implying a functional link between IKKα expression and c-Myc levels in humans in vivo. Of note, none of the cancer variants showed a negative correlation (Suppl. Figure S4).

### Effect of the IKKα target site of c-Myc on apoptosis and proliferation

Based on the notion that IKKα-mediated phosphorylation of serine-67 and -71 of c-Myc stabilize the oncogene, we hypothesized that this might have an impact on apoptosis or proliferation of cells. To test that, we analyzed stable transfectant DU145 cell clones expressing the phospho-mimetic c-MycEE, the non-phosphorylatable c-MycAA or c-Myc.

**Fig. 6:**
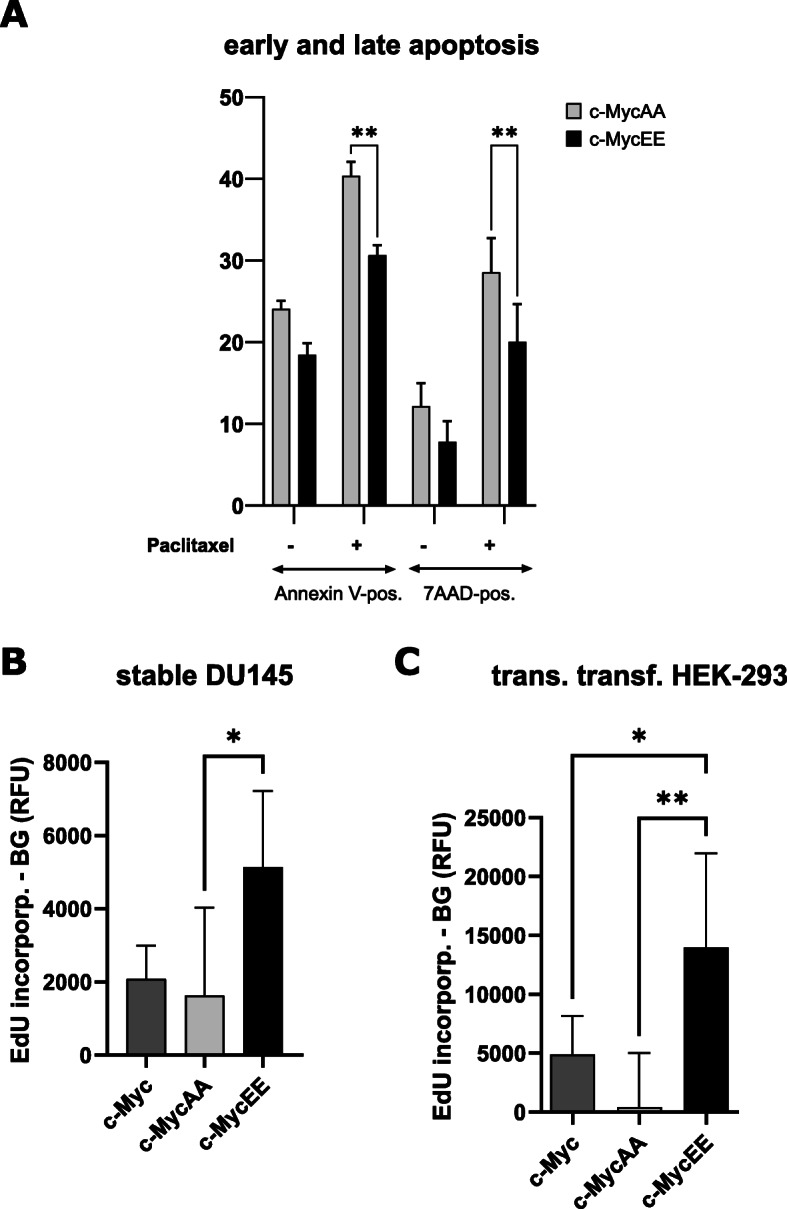
Effect of c-Myc phosphorylation mutants on apoptosis and proliferation. **a** Expression constructs of c-Myc with serines 67/71 mutated to alanines (preventing phosphorylation of these residues: c-MycAA), or mutated to glutamates (c-MycEE, mimicking IKKα-mediated phosphorylation) were stably transfected into DU145 cells. These cells were either left untreated or treated with 10 nM paclitaxel to stimulate apoptosis, followed by labeling with Annexin V (for early apoptotic cells) or 7AAD (for late apoptosis and necrosis) and measured by flow cytometry (*n* = 3, mean  % positive cells +/−SD). **b** Proliferation of stable DU145 transfectants expressing c-Myc, c-MycAA or c-MycEE was assessed by culturing of cells in presence of 6 μM EdU for 4 h (to label cells in S-phase of the cell cycle). EdU was labeled by click-chemistry with TAMRA and the fluorescence was quantified on a plate reader with excitation at 546 nm and emission at 580 nm (*n* = 5, mean +/− standard deviation). **c** HEK-293 cells were transiently transfected with the c-Myc variants, followed by EdU-labeling of proliferating cells and quantification as described in (**b**). *n* = 6, Mean values are plotted with error bars indicating standard deviation

Treating these cells with the chemotherapeutic drug paclitaxel revealed that c-MycEE expressing cells were protected from apoptosis when compared with c-MycAA-expressing cells both for early apoptosis (detected via Annexin V-binding) and for late apoptosis or necrosis (as assessed by 7-AAD staining, Fig. 6a). On the other hand, cell proliferation as determined by EdU incorporation during DNA-synthesis was significantly higher for c-MycEE transfectants (Fig. 6b). These findings were supported by experiments with transiently transfected HEK-293 cells (Fig. 6c).

**Fig. 7:**
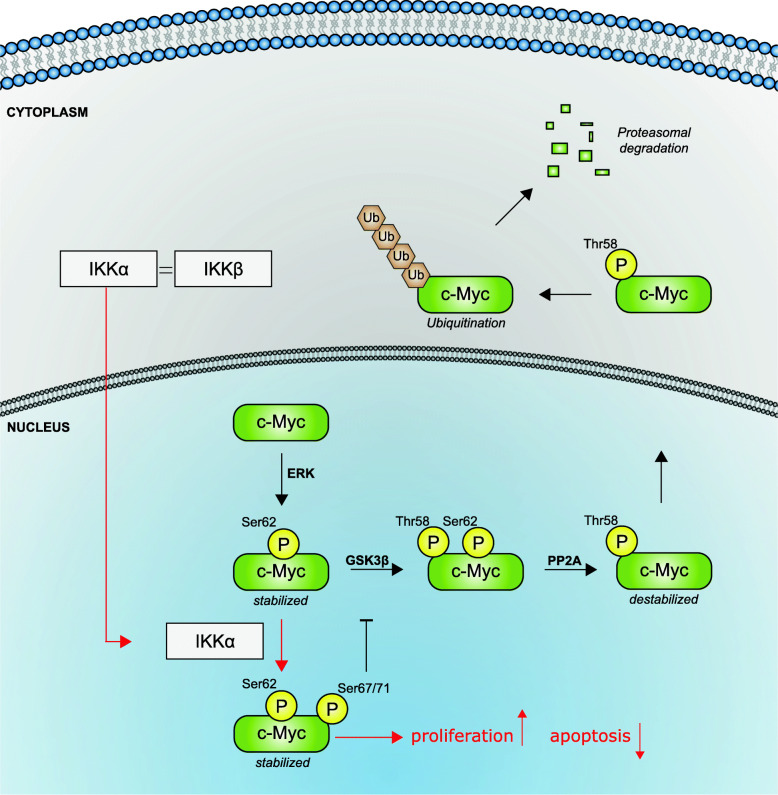
IKKα – c-Myc activation model. c-Myc is phosphorylated by ERK, GSK3β or other kinases on serine-62. GSK3β further phosphorylates c-Myc on threonine-58 leading to dephosphorylation of serine-62 and subsequent ubiquitination and proteasomal degradation. IKKα interacts with c-Myc and phosphorylates it at serine-67 and serine-71 presumably in the nucleus, leading to a subsequent inhibition of GSK3β-mediated phosphorylation of threonine-58 followed by stabilization and increased transcriptional activity of c-Myc leading to an increase in proliferation and a decrease in apoptosis

Taken together, our results support a model in which IKKα interacts with c-Myc, phosphorylating it at serines-67/71 most likely within the nucleus, the predominant localization of c-Myc, leading to a subsequent inhibition of GSK3β-mediated phosphorylation and a concomitant stabilization of c-Myc. A consequence of that is an elevated transcriptional activity of c-Myc followed by an increase in cell proliferation and a reduced susceptibility to apoptosis (Fig. 7).

## Discussion

In this study, we investigated effects of the IκB kinases, IKKα and IKKβ, on the oncoprotein c-Myc. We showed that both kinases can interact with c-Myc and are able to phosphorylate the N-terminal end of c-Myc at serine-67 and serine-71 (the motif SGLCS). However, in vivo only IKKα seemed to affect the phosphorylation of c-Myc at threonine-58, thereby enhancing its stability and transcriptional activity. This is in line with immunofluorescence staining of mouse prostate sections showing that expression of IKKα correlates with the c-Myc protein levels, as well as with human studies showing a positive correlation between IKKα and c-Myc expression in several different cancer types. In contrast to IKKβ, which resides mainly in the cytoplasm, IKKα shuttles between the cytoplasm and the nucleus [7–9, 11], and has a higher tendency to be present in the nucleus, where it can interact with c-Myc, which localizes nearly exclusively to the nucleus. While our data indicate that not only IKKα but also IKKβ can serve as kinase for c-Myc, the lack of an effect of the CRISPR/Cas9-mediated IKKβ knockout on threonine-58 phosphorylation and activity of c-Myc would argue against a significant role of that kinase for regulation of c-Myc. However, it cannot be excluded that minor amounts of c-Myc, occurring in the cytosol, are phosphorylated by IKKβ, which might have additional effects.

In a previous report, it has been shown that expression of both, IKKα and IKKβ, correlates with c-Myc expression in breast cancer cells and that both kinases promote c-Myc protein stability in these cells, while only IKKα could interact with c-Myc [16]. The authors had used an IKK-inhibitor (Bay11–0782), which had been frequently used for inhibition of IKKs in earlier times, but which later has been demonstrated to be very unspecific and to induce apoptosis independent from inhibition of NF-κB activation [17, 42, 43]. In our study, we used BMS-345541, which has been proven highly specific for IKKα (with an IC50 of 4 μM) and IKKβ (IC50: 0.3 μM) [44]. BMS-345541 treatment at a concentration, where it inhibits both kinases, significantly increased phosphorylation of threonine-58 on c-Myc while serine-62 phosphorylation was unaffected. In order to exclude off-target effects of the kinase inhibitors and for a clear discrimination between IKKα and IKKβ, we generated knockout cell lines for these two kinases using CRISPR/Cas9 technology. With that approach, we could finally identify IKKα as the IκB kinase responsible for regulating c-Myc threonine-58 phosphorylation and subsequently its stability and transcriptional activity. Our results strongly suggest a model in which IKKα interacts with c-Myc, followed by phosphorylation of serine-67 and serine-71, which are in close vicinity to the GSK3β target site. We assume that this leads to subsequent hampering of GSK3β-dependent phosphorylation, probably by changing the GSK3β recognition domain via the addition of negative charges through the phosphate residues added by IKKα. A similar mechanism was shown for Aurora B kinase (AURKB), which promotes c-Myc stability by phosphorylating serine-67 and counteracting GSK3β-directed threonine 58 phosphorylation [45].

In principle, it could be possible that IKKα regulates c-Myc activity at distinct genomic regions. This has been shown for SMRT or p27 (CDKN1B), which are bound and phosphorylated by IKKα at specific gene promoters, leading to their export from the nucleus [46, 47]. Whether c-Myc phosphorylation by IKKα influences certain genes specifically remains to be elucidated, but the fact that we could detect a global change in c-Myc phosphorylation would argue for a more general role of this process in upregulating c-Myc activity and could explain, why inflammatory states favor malignant transformations or the progress of cancer development. This notion is supported by our findings that a c-Myc-mutant mimicking IKKα-phosphorylation at serines 67/71 exhibits a higher rate of DNA-synthesis and lower apoptosis in presence of a cytotoxic drug, which is also in line with several studies showing pro-tumorigenic roles of IKKα in various cancers [12, 14–16, 48–52].

## Conclusion

In this study, we demonstrate that transcriptional activity of c-Myc is increased by IKKα via phosphorylation at serines 67/71, which hinders threonine-58 phosphorylation and thus degradation. This represents another link between inflammatory signaling molecules and cancer, in addition to the known transcriptional upregulation of c-Myc by NF-κB [53].

## Supplementary Information

**Additional file 1: Figure S1.** FRET microscopy demonstrating interaction of c-Myc with IKKα, and to a minor extent with IKKβ. (A) HEK-293 cells were transfected with red-fluorescent-protein tagged c-Myc (or red fluorescent protein alone, neg. control) in combination with EGFP-tagged IKKα, EGFP-tagged IKKβ (or EGFP alone, neg. control). 3-Filter FRET microscopy was performed for donor (EGFP), acceptor (red fluorescent protein) and the raw FRET signal (donor excitation and acceptor emission), followed by calculation of corrected FRET images eliminating the spectral bleed-through (FRETcor.) and computation of normalized FRET images (FRETcor.-images normalized to expression levels). (B) Normalized FRET images as shown in (A) were used to calculate mean values of the normalized FRET signal for comparison of the samples as indicated (*n* = 8).

**Additional file 2: Figure S2.** IKKα knockout affects phosphorylation of c-Myc at threonine-58. Western blot analysis for threonine-58 (T58), serine-62 (S62) phosphorylation of c-Myc and GSK3β of cell extracts of control or IKKα or IKKβ knockout cells in absence or presence of the IKK-inhibitor BMS-345541. Cells were additionally treated with TNFα, indicating a NF-κB-independent effect.

**Additional file 3: Figure S3.** Turnover of c-Myc variants as determined by protein synthesis blockade via cycloheximide. (A) Stable DU145 transfectants expressing c-MycAA or c-MycEE were cultured for different time periods in presence of 60 μg/ml cycloheximide (CHX) to block protein synthesis, followed by cell lysis, SDS-PAGE and Western blotting for c-Myc. (B) Optical pulse-chase of Dendra2-labeled c-Myc, c-MycEE and c-MycAA mutants. Decrease of the red fluorescence intensity after conversion of green Dendra2 to the red fluorescent form due to degradation of photoconverted proteins. Cells expressing wild-type c-Myc tagged with Dendra2, which had been fixed by paraformaldehyde served as controls for potential bleaching of the red fluorescent form by repetitive imaging (*n* = 4, mean values ± standard deviation).

**Additional file 4: Figure S4.** IKKα and c-Myc expression correlates in several different cancer types. (A) Pearson’s correlation between the expression values in Transcript Per Million of IKKα (CHUK) and c-Myc (MYC) in several different types of human cancers from the TCGA study. The plot scale was transformed to a log10 scale. ACC: Adrenocortical carcinoma; BLCA: Bladder Urothelial Carcinoma; BRCA: Breast invasive carcinoma; COAD: Colon adenocarcinoma; READ: Rectum adenocarcinoma; ESCA: Esophageal carcinoma; KICH: Kidney Chromophobe; KIRC: Kidney renal clear cell carcinoma; KIRP: Kidney renal papillary cell carcinoma; LIHC: Liver hepatocellular carcinoma; LUAD: Lung adenocarcinoma; LUSC: Lung squamous cell carcinoma; MESO: Mesothelioma; OV: Ovarian serous cystadenocarcinoma, PAAD: Pancreatic adenocarcinoma; PCPG: Pheochromocytoma and Paraganglioma; PRAD: Prostate adenocarcinoma; SKCM: Skin Cutaneous Melanoma; STAD: Stomach adenocarcinoma; UVM: Uveal Melanoma; Lines represent the linear regression analysis; r is the Spearman correlation coefficient, *p*-values indicate statistical significance of positive correlation according to Spearman. (B) Left panel: Correlation of c-Myc and IKKα expression in mouse prostates. Each dot represents a small region of the prostate epithelium normalized to the nuclear stain (DAPI). Right panel: Expression levels of IKKα and c-Myc in wildtype and Hi-MYC mice depicted as violin blots. For statistical analysis a Kruskal-Wallis test followed by Dunn’s multiple comparison was performed. Dotted lines represent the median value. *p*-values: ‘****’ for *p* < 0.0001.

## Data Availability

The data and materials of the study are available from the corresponding author upon reasonable request.

## Abbreviations

CDK4: Cyclin-dependent kinase 4
EGFR: Epidermal growth factor receptor
FRET: Fluorescence energy transfer
GSK3β: Glycogen synthase kinase 3β
HLH: Helix-loop-helix
IKK: IκB kinase
LZ: Leucine zipper
Max: Myc-associated factor
MMP-9: Matrix metallopeptidase 9
NEMO: NF-κB essential modulator
NF-κB: Nuclear factor kappa-light chain-enhancer of activated B cells
S62: Serine-62
T58: Threonine-58
VEGF-A: Vascular endothelial growth factor A
