# Supplementary figures and images for: The inflammatory kinase IKKα phosphorylates and stabilizes c-Myc and enhances its activity
Source: Mol Cancer. 2021 Jan 18;20:16. doi: 10.1186/s12943-021-01308-8 (PMC7812655; doi:10.1186/s12943-021-01308-8)

**A**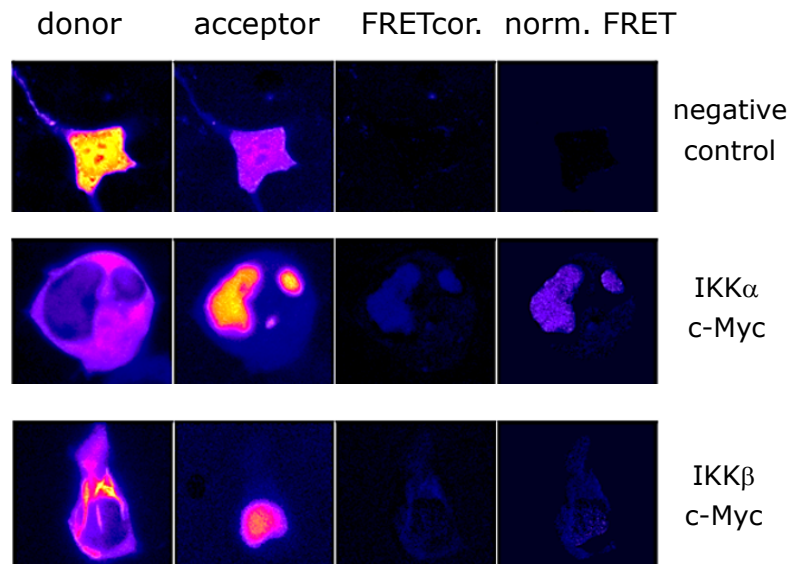**B**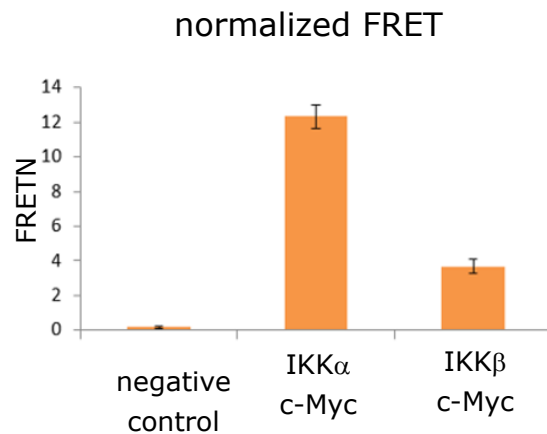

Supplement: Supplementary file 1 — Additional file 1: Figure S1. FRET microscopy demonstrating interaction of c-Myc with IKKα, and to a minor extent with IKKβ. (A) HEK-293 cells were transfected with red-fluorescent-protein tagged c-Myc (or red fluorescent protein alone, neg. control) in combination with EGFP-tagged IKKα, EGFP-tagged IKKβ (or EGFP alone, neg. control). 3-Filter FRET microscopy was performed for donor (EGFP), acceptor (red fluorescent protein) and the raw FRET signal (donor excitation and acceptor emission), followed by calculation of corrected FRET images eliminating the spectral bleed-through (FRETcor.) and computation of normalized FRET images (FRETcor.-images normalized to expression levels). (B) Normalized FRET images as shown in (A) were used to calculate mean values of the normalized FRET signal for comparison of the samples as indicated (n = 8). [file 12943_2021_1308_MOESM1_ESM.pdf]

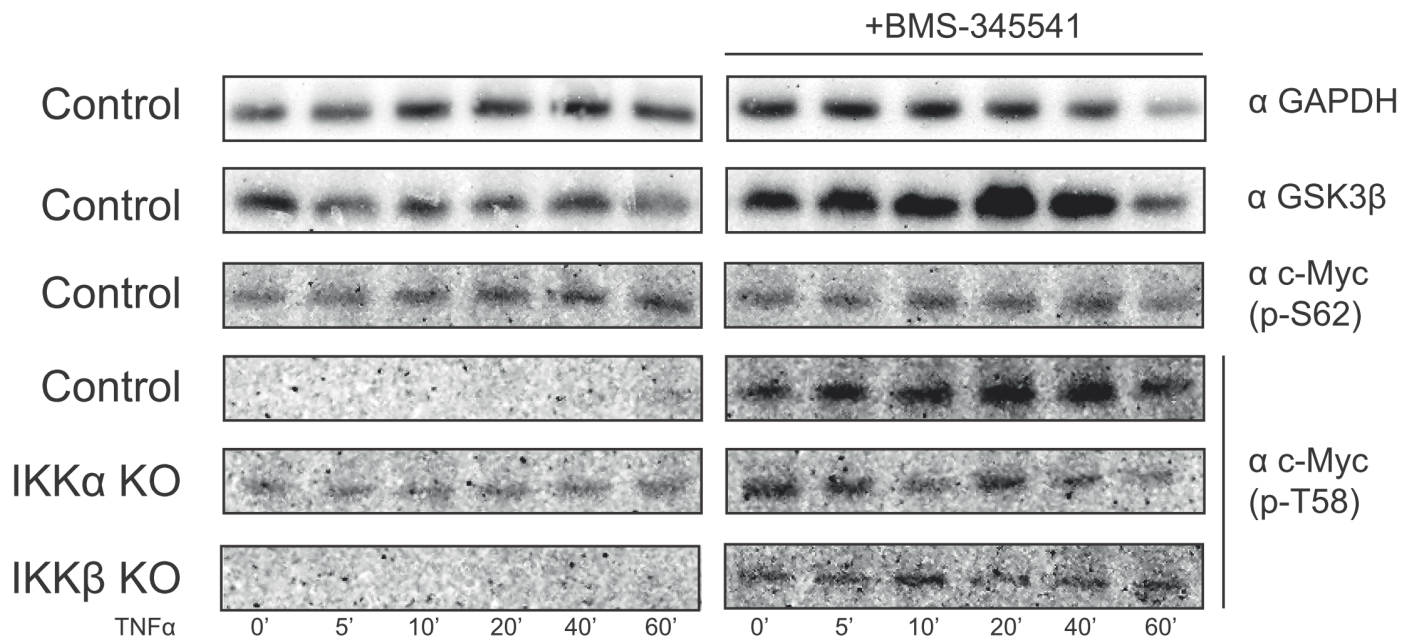

Supplement: Supplementary file 2 — Additional file 2: Figure S2. IKKα knockout affects phosphorylation of c-Myc at threonine-58. Western blot analysis for threonine-58 (T58), serine-62 (S62) phosphorylation of c-Myc and GSK3β of cell extracts of control or IKKα or IKKβ knockout cells in absence or presence of the IKK-inhibitor BMS-345541. Cells were additionally treated with TNFα, indicating a NF-κB-independent effect. [file 12943_2021_1308_MOESM2_ESM.pdf]

A

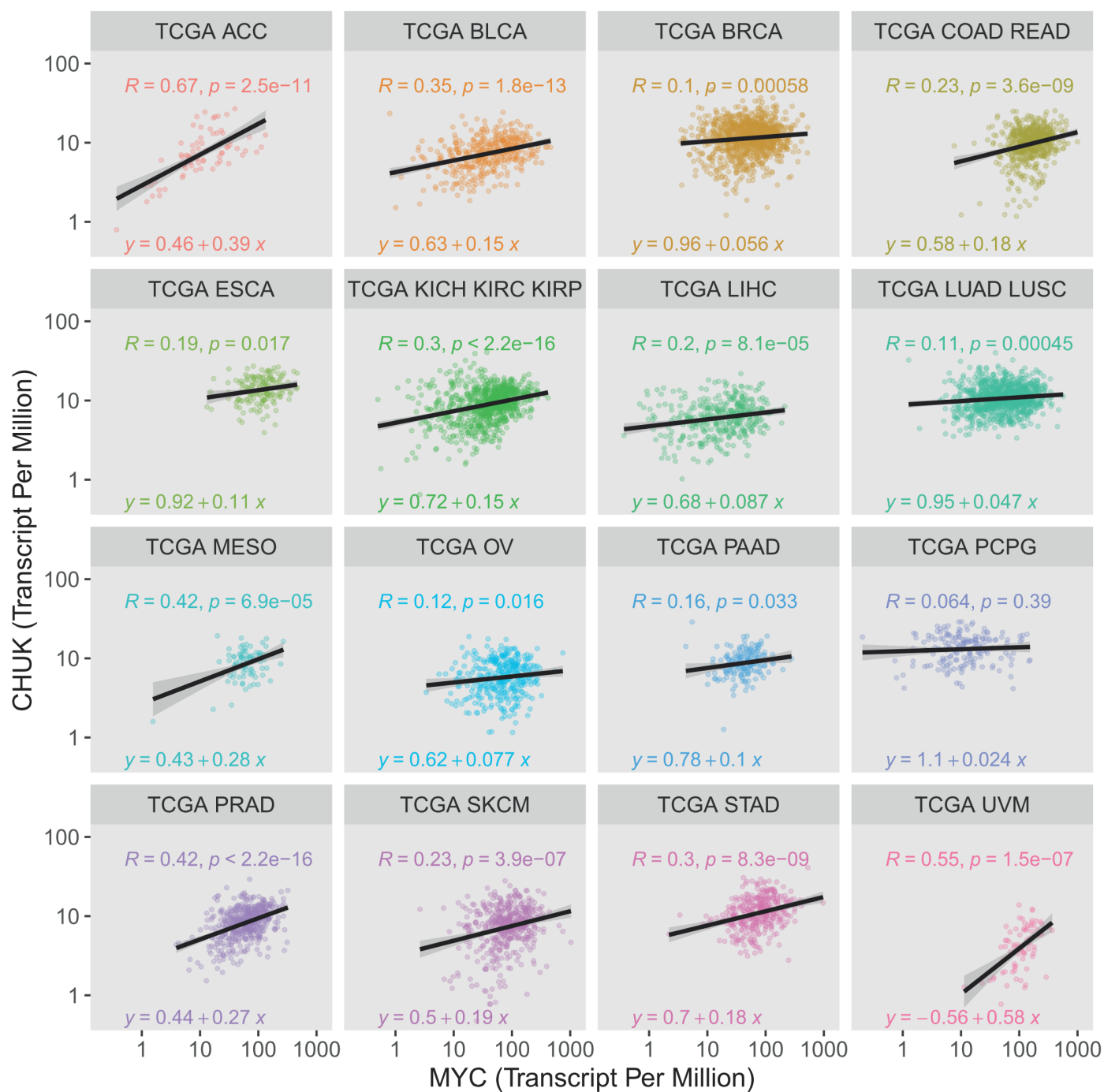

B

## Wildtype and Hi-MYC mice

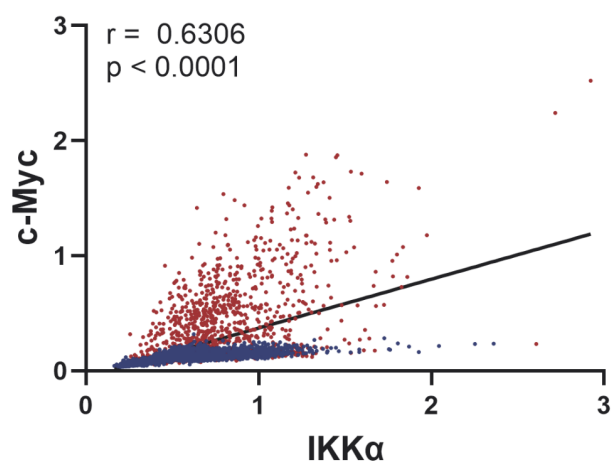

## Wildtype and Hi-MYC mice

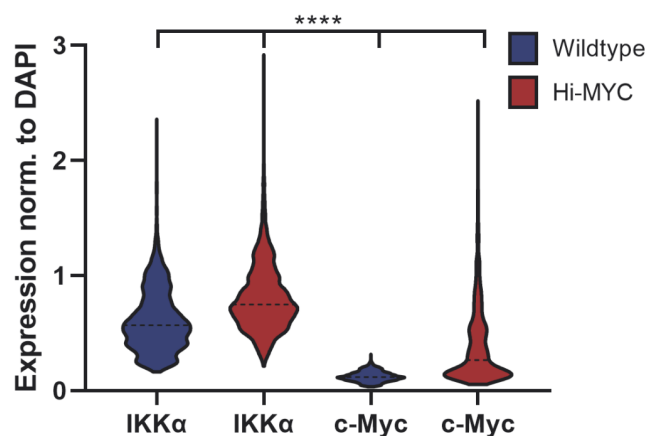

Supplement: Supplementary file 4 — Additional file 4: Figure S4. IKKα and c-Myc expression correlates in several different cancer types. (A) Pearson’s correlation between the expression values in Transcript Per Million of IKKα (CHUK) and c-Myc (MYC) in several different types of human cancers from the TCGA study. The plot scale was transformed to a log10 scale. ACC: Adrenocortical carcinoma; BLCA: Bladder Urothelial Carcinoma; BRCA: Breast invasive carcinoma; COAD: Colon adenocarcinoma; READ: Rectum adenocarcinoma; ESCA: Esophageal carcinoma; KICH: Kidney Chromophobe; KIRC: Kidney renal clear cell carcinoma; KIRP: Kidney renal papillary cell carcinoma; LIHC: Liver hepatocellular carcinoma; LUAD: Lung adenocarcinoma; LUSC: Lung squamous cell carcinoma; MESO: Mesothelioma; OV: Ovarian serous cystadenocarcinoma, PAAD: Pancreatic adenocarcinoma; PCPG: Pheochromocytoma and Paraganglioma; PRAD: Prostate adenocarcinoma; SKCM: Skin Cutaneous Melanoma; STAD: Stomach adenocarcinoma; UVM: Uveal Melanoma; Lines represent the linear regression analysis; r is the Spearman correlation coefficient, p-values indicate statistical significance of positive correlation according to Spearman. (B) Left panel: Correlation of c-Myc and IKKα expression in mouse prostates. Each dot represents a small region of the prostate epithelium normalized to the nuclear stain (DAPI). Right panel: Expression levels of IKKα and c-Myc in wildtype and Hi-MYC mice depicted as violin blots. For statistical analysis a Kruskal-Wallis test followed by Dunn’s multiple comparison was performed. Dotted lines represent the median value. p-values: ‘****’ for p < 0.0001. [file 12943_2021_1308_MOESM4_ESM.pdf]
