# Supplementary material for: The inflammatory kinase IKKα phosphorylates and stabilizes c-Myc and enhances its activity
Source: Mol Cancer. 2021 Jan 18;20:16. doi: 10.1186/s12943-021-01308-8 (PMC7812655; doi:10.1186/s12943-021-01308-8)
Supplement: Supplementary file 3 — Additional file 3: Figure S3. Turnover of c-Myc variants as determined by protein synthesis blockade via cycloheximide. (A) Stable DU145 transfectants expressing c-MycAA or c-MycEE were cultured for different time periods in presence of 60 μg/ml cycloheximide (CHX) to block protein synthesis, followed by cell lysis, SDS-PAGE and Western blotting for c-Myc. (B) Optical pulse-chase of Dendra2-labeled c-Myc, c-MycEE and c-MycAA mutants. Decrease of the red fluorescence intensity after conversion of green Dendra2 to the red fluorescent form due to degradation of photoconverted proteins. Cells expressing wild-type c-Myc tagged with Dendra2, which had been fixed by paraformaldehyde served as controls for potential bleaching of the red fluorescent form by repetitive imaging (n = 4, mean values ± standard deviation). [file 12943_2021_1308_MOESM3_ESM.pdf]

A

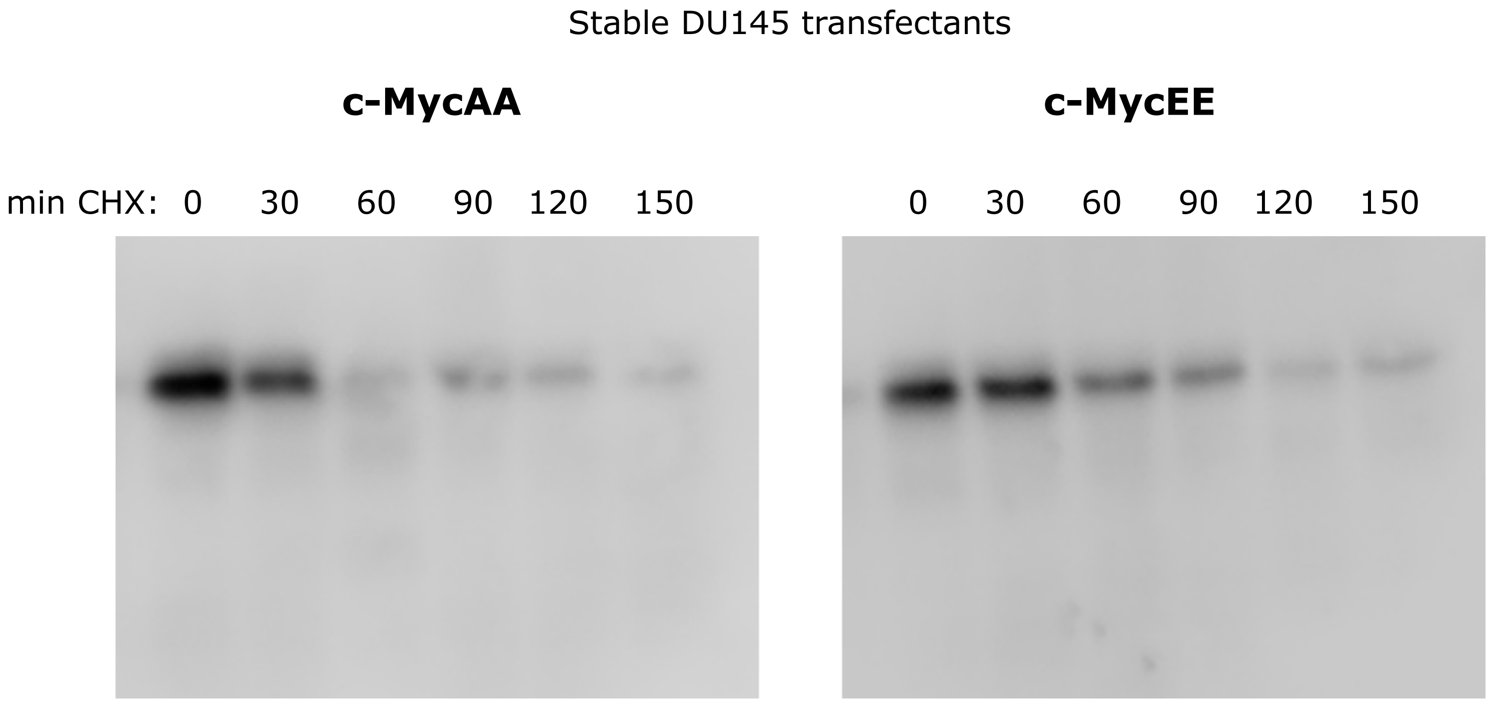
Stable DU145 transfectants
c-MycAA
c-MycEE
min CHX: 0 30 60 90 120 150
0 30 60 90 120 150

B

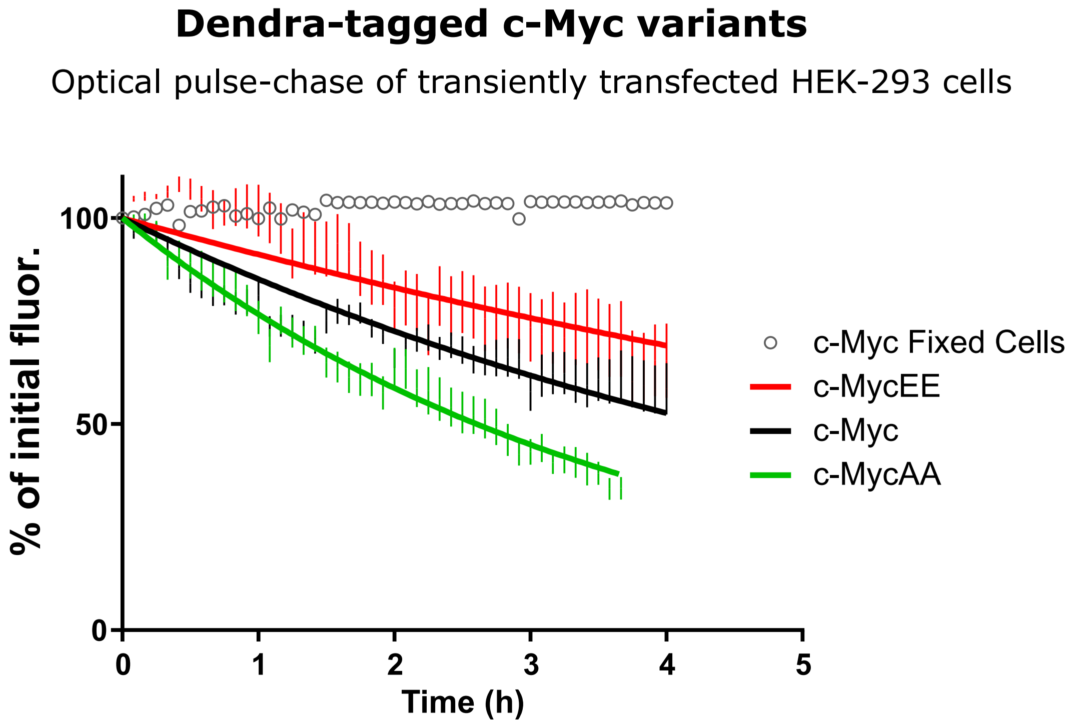
Dendra-tagged c-Myc variants
Optical pulse-chase of transiently transfected HEK-293 cells
% of initial fluor.
100
50
0
0
1
2
3
4
5
Time (h)
c-Myc Fixed Cells
c-MycEE
c-Myc
c-MycAA
